# Editorial: Engineering antibodies and bacteriophages to manage and diagnose microbial infections

**DOI:** 10.3389/fmicb.2025.1567335

**Published:** 2025-02-18

**Authors:** Jamshid Tanha, Kenneth K. S. Ng, Chang-Chun Ling

**Affiliations:** ^1^Human Health Therapeutics Research Centre, Life Sciences Division, National Research Council Canada, Ottawa, ON, Canada; ^2^Department of Biochemistry, Microbiology and Immunology, Faculty of Medicine, University of Ottawa, Ottawa, ON, Canada; ^3^Department of Chemistry and Biochemistry, Faculty of Science, University of Windsor, Windsor, ON, Canada; ^4^Department of Chemistry, Faculty of Science, University of Calgary, Calgary, AB, Canada

**Keywords:** antibody engineering, bacteriophage engineering, antimicrobial treatment, diagnosis of microbial infections, antimicrobial resistance

There is an urgent and growing need for novel and effective antimicrobial agents to combat the increasing number of pathogens resistant to multiple classes of antimicrobial chemotherapeutics. Exploring treatments and diagnostics for pathogens using the complementary strengths offered by antibodies and bacteriophages promises to provide novel solutions to meet the formidable challenges posed by emerging pathogens and well-known pathogens where resistance to antimicrobial chemotherapeutics is becoming a growing threat. These approaches build upon a broad and ever-expanding variety of antibody formats that include monoclonal antibodies, single-chain Fvs and single-domain antibodies, as well as diverse natural and engineered bacteriophages, chemical conjugates and phage-derived enzymes.

This Research Topic focuses on studies that contribute to the exploration and development of some of the emerging tools based on antibodies and bacteriophages to manage and diagnose microbial infections.

Hussack et al. describe a structure-guided approach for designing a potent *Clostridioides difficile* toxin A inhibitor as a potential therapeutic for *C. difficile* infections. Crystal structures of two camelid nanobodies, A20 and A26, bound to fragments of *C. difficile* toxin A revealed the precise geometry of the nanobodies in relation to each other and to the binding sites for cell-surface carbohydrate receptors. Based on these observations, the authors created a biparatopic fusion protein with A20 at the N-terminus, followed by a linker of precise size and A26 at the C-terminus. This A20-A26 fusion protein showed improved affinity and a dramatic increase in toxin A neutralization potency—up to ~3,000-fold—compared to the unfused A20 and A26 nanobodies. The importance of molecular geometry and the benefit of simultaneously engaging the epitopes for both A20 and A26 were strikingly illustrated by the 29,000-fold higher neutralization potency of A20-A26 compared to A26-A20, a biparatopic fusion control with A26 at the N-terminus and A20 at the C-terminus. These results underscore the power of structure-based design in generating highly potent antibody-based reagents that exploit the simultaneous binding of multiple paratopes on an antigen.

In their original research article, Cai et al. report the isolation and comprehensive characterization of a lytic phage that displayed a broad host range across various *Vibrio* species and stability over a wide range of temperature and pH levels. These properties help to make this a potentially practical and effective new therapeutic against vibriosis, a bacterial disease that causes significant mortality and economic losses in aquaculture. The authors found no evidence of antibiotic resistance or virulence genes in the phage's genome, thus reducing safety concerns for use in biocontrol applications.

Tian et al. describe the isolation, characterization and therapeutic evaluation of a new lytic phage targeting *Acinetobacter baumannii*, an opportunistic pathogen resistant to currently available antibiotics. In an *in vivo* model, the phage increased the rates of survival for larvae infected with *A. baumannii*, thus helping to demonstrate its therapeutic potential. Moreover, the phage genome was found to lack any virulence-related or antibiotic-resistance genes, enhancing its appeal as an alternative to antibiotics for treating *A. baumannii* infections. Peters et al. present a different phage-based approach toward developing potential therapeutics against *A. baumannii* infections. Phage depolymerases, which degrade the bacterial capsule polysaccharide layer, show promise by sensitizing the bacteria to phage infections, the effects of selected antibiotics, and serum killing. Peters et al. analyzed previously published sequences and predicted structures of tail fiber proteins from *A. baumannii* phages, identifying a diverse range of domains associated with depolymerase function. This work expands our understanding of phage depolymerases and enables researchers to better exploit these enzymes for future therapeutic applications to combat the antimicrobial resistance crisis posed by *A. baumannii*.

A novel approach to use the power of machine learning (ML) to help harness the information from vast and ever-expanding databases of genomic and metagenomic sequences is described by the original research article from Albin et al.. The open-source PhageScanner ML pipeline includes modules for data curation, model training and prediction/data visualization to help classify and shed light on the functions and properties of proteins encoded by open reading frames in phage genomes or metagenomic data. The effectiveness of this approach was demonstrated by applying the pipeline to identify phage virion proteins from six previously uncharacterized phage genomes.

In an opinion article, Szymanski explores how bacteriophages and their unique components provide limitless resources for exploitation. After providing a concise overview of the diverse contributions and applications of phages—such as informing our understanding of biological and chemical processes, enabling key historical discoveries, and their use in disease therapy and detection, as well as in antibody phage display—the author focuses on three emerging trends. These include exploiting phages for (i) glyco-tool discovery, (ii) glycan display, and (iii) vaccine development. Szymanski emphasizes that phages will serve as a constant source of reagents for glycobiologists, become increasingly utilized in glycan display platforms to better understand carbohydrate-protein binding specificities and notes that enveloped phages have the potential to replace lipid nanoparticles, virus-like particles and outer membrane vesicles for many vaccine applications.

In a review more directly focused upon therapeutic applications, Subramanian examines the emerging roles of phage-based therapeutics in combating antibiotic resistance. The review highlights the advantages of phage-based therapies over traditional antibiotics, the synergies of combining phage and antibiotics, the potential of phage-derived proteins as antimicrobial agents and formulation strategies in phage therapy. It also discusses recent progress in the pre-clinical and clinical studies of phage-based therapeutics targeting clinically significant pathogens, along with the current challenges and opportunities of advancing phage therapy.

The diversity of topics addressed by the engineering of novel therapeutics and diagnostics based on antibodies and phages helps to highlight some of the recent advances and emerging directions in this very active and promising research area for combatting the growing challenges of antimicrobial resistance and emerging infectious diseases.

